# Do Poor Diet and Lifestyle Behaviors Modify the Genetic Susceptibility to Impulsivity in the General Population?

**DOI:** 10.3390/nu15071625

**Published:** 2023-03-27

**Authors:** Tian Xie, Lizanne J. S. Schweren, Henrik Larsson, Lin Li, Ebba Du Rietz, Jan Haavik, Liv Grimstvedt Kvalvik, Berit Skretting Solberg, Kari Klungsøyr, Harold Snieder, Catharina A. Hartman

**Affiliations:** 1 Interdisciplinary Center Psychopathology and Emotion Regulation (ICPE), Department of Psychiatry, University Medical Center Groningen, University of Groningen, Hanzeplein 1, 9700 RB Groningen, The Netherlands; 2 Department of Epidemiology, University Medical Center Groningen, University of Groningen, Hanzeplein 1, 9700 RB Groningen, The Netherlands; 3 School of Medical Sciences, Örebro University, 70172 Örebro, Sweden; 4 Department of Medical Epidemiology and Biostatistics, Karolinska Institutet, 17165 Stockholm, Sweden; 5 Department of Biomedicine, University of Bergen, 5020 Bergen, Norway; 6 Bergen Center for Brain Plasticity, Division of Psychiatry, Haukeland University Hospital, 5012 Bergen, Norway; 7 Department of Global Public Health and Primary Care, University of Bergen, 5020 Bergen, Norway; 8 Child- and Adolescent Psychiatric Outpatient Unit, Hospital Betanien, 5143 Bergen, Norway; 9 Division of Mental and Physical Health, Norwegian Institute of Public Health, 5015 Bergen, Norway

**Keywords:** dietary habits, lifestyle behaviors, genetic susceptibility, diet-gene interaction, impulsivity

## Abstract

The present study investigated whether an unhealthy diet and other lifestyle behaviors may modify the genetic susceptibility to impulsivity. A total of 33,047 participants (mean age = 42.1 years, 59.8% females) from the Dutch Lifelines cohort were included. Each diet index and other lifestyle behaviors were tested for their interactions on the effect on the attention-deficit/hyperactivity disorder (ADHD) polygenic risk score (PRS) on impulsivity using a linear regression model with adjustment for covariates. The ADHD PRS was significantly associated with impulsivity (B = 0.03 (95% CI: 0.02, 0.04); *p* = 2.61 × 10^−9^). A poorer diet, a higher intake of energy, and a higher intake of fat were all associated with higher impulsivity, and a high intake of energy amplified the effect of ADHD PRS on impulsivity (e.g., for the interaction term of ADHD PRS and highest tertile on intake of energy, B = 0.038 (95% CI: 0.014, 0.062); *p* = 0.002. The other lifestyle factors, namely short and long sleep duration, current and past smoking, higher alcohol intake, and more time spent on moderate-to-vigorous physical activity were associated with higher impulsivity, but no interaction effect was observed. In conclusion, we found that a high intake of energy exacerbated the genetic susceptibility to impulsivity. Our study helps to improve our understanding of the role of diet and genetic factors on impulsivity.

## 1. Introduction

Impulsivity is a complex, multidimensional trait that may be defined as ‘a predisposition toward rapid, unplanned reactions to internal or external stimuli, with diminished regard to the negative consequences that such reactions may have for the impulsive individual or others’ [1,2]. The lifetime prevalence of self-reported impulsivity was found to be 16.9% in a large national sample of the United States population [3]. Impulsivity is one of core symptoms of attention-deficit/hyperactivity disorder (ADHD) that is a persistent neurodevelopmental disorder affecting 2.5% of adults worldwide [4]. Furthermore, individuals with excessive or maladaptive impulsivity are at increased risk of serious consequences, including aggressive and criminal behavior, as well as developing psychiatric disorders, such as bipolar spectrum disorders and substance abuse disorders [1]. These problems, along with their considerable societal costs [5], justify research on impulsivity to better understand its complicated etiology, particularly the modifiable factors that may be potential targets in preventive interventions.

Both genetic and environmental factors influence impulsivity. A meta-analysis of twin, family, and adoption studies demonstrated that approximately 50% of the variance in impulsivity was explained by genetic influences [6]. A genome-wide association study (GWAS) on impulsive personality traits in up to 22,861 adults identified two genome-wide significant loci [7]. However, most of our knowledge on impulsivity genes comes from studies on ADHD [8,9], which have shown a strong genetic correlation with impulsivity [7,10]. The latest GWAS meta-analysis of ADHD on 38,691 individuals with ADHD and 186,843 controls identified 27 genome-wide significant loci, which is more than twice the number previously reported [9]. Previous studies showed that a polygenic risk of ADHD was consistently associated with trait impulsivity at ages spanning from early childhood to adolescence [11,12]. These studies used earlier GWASs [8,13] to calculate the polygenic risk of ADHD, and as ADHD genetic studies keep increasing their sample sizes, an updated polygenic risk of ADHD using the latest GWAS is expected to have a better prediction accuracy for impulsivity.

Previous research has also revealed associations between impulsivity and environmental factors, such as lifestyle choices. For instance, a cross-sectional study of 51,368 adult participants found that impulsivity was positively associated with energy intake and negatively associated with diet quality [14]. A longitudinal study showed that a diet high in ‘junk food’ in early childhood was associated with increased hyperactivity at age seven [15]. Furthermore, adhering to multiple lifestyle recommendations in early adolescence has been shown to have strong associations with a decreased incidence of ADHD until age 14 [16]. 

Furthermore, etiological theories highlight interactions between genes and the environment underlying impulsivity [17]. Twin studies have shown that genetic factors play a more prominent role in ADHD development in the presence of a diet rich in sugar and unhealthy foods, which suggests the existence of diet-gene interactions in ADHD symptoms [18]. Several studies also used a candidate gene approach to explore the gene-environment interaction in ADHD or impulsivity. For example, two studies suggested that maternal prenatal smoking, as well as maternal use of alcohol during pregnancy modified the impact of the dopamine transporter gene (DAT) on ADHD, i.e., DAT was associated with ADHD only when the children had exposure to maternal prenatal smoking or maternal alcohol drinking during the pregnancy [19,20]. However, the selection of appropriate candidate genes in these studies assumes solid knowledge of the specific underlying biological mechanisms that remains rather limited. Furthermore, recent genetic studies [7,9] suggest that the genetic architecture of ADHD and impulsivity are highly complex and polygenic, i.e., they are influenced by thousands of common variants with minor effects rather than by a few variants of large effects. In light of these limitations, GxE research has transitioned from the candidate gene approach toward the polygenic risk score (PRS) approach that aggregates the effects of individual common genetic variants on traits [17]. Nonetheless, few studies have addressed the diet-gene interaction on impulsivity or ADHD using the PRS approach, and research exploring interactions between genetic factors and a wide range of lifestyle factors in impulsivity or ADHD is also lacking [21].

Therefore, in our study, we investigated whether an unhealthy diet and other lifestyle behaviors (i.e., physical activity, sleep, smoking, drinking) modify the genetic susceptibility to impulsivity, a symptom of ADHD, using a calculated PRS based on the most recent meta-GWAS results on ADHD.

## 2. Materials and Methods

### 2.1. Participants

The Lifelines cohort study (Lifelines) is a multi-disciplinary prospective population-based cohort study examining the health and health-related behaviors of 167,729 persons living in the north of the Netherlands, in a unique three-generation design [22]. It employs a broad range of investigative procedures in assessing the biomedical, socio-demographic, behavioral, physical, and psychological factors that contribute to the health and disease of the general population, with a particular focus on multi-morbidity and complex genetics. Between 2006 and 2013, a large number of general practitioners (GPs) were involved and invited all of their listed patients aged 25–50 years. Willing respondents and their family members (children, parents, partners) were asked to participate. 

**Figure 1:**
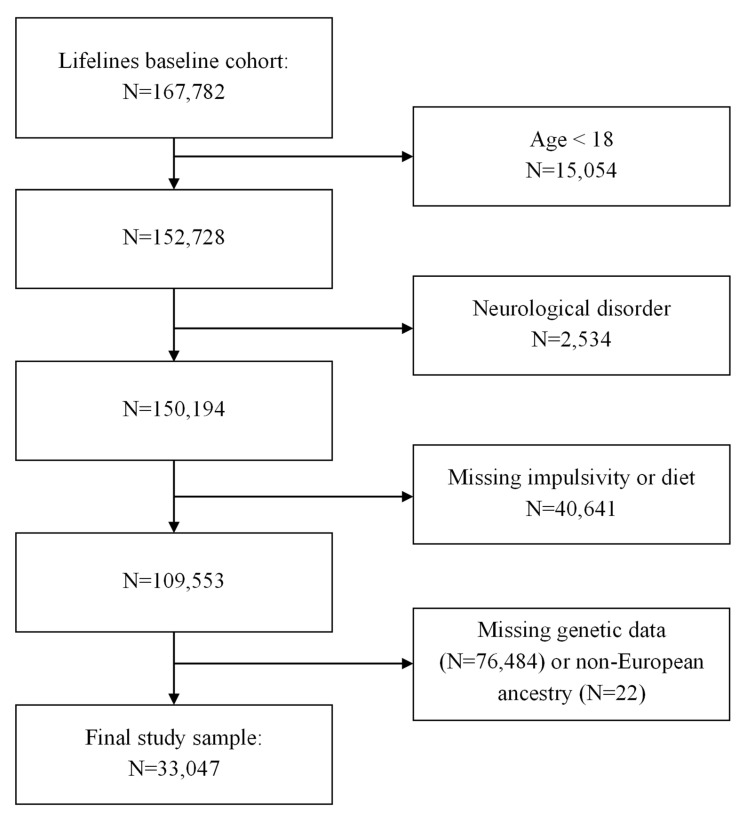
Flowchart of the selection of the study participants.

For the current study, baseline data were used applying the following exclusion criteria: (a) age < 18; (b) self-reported diagnosis of any neurological disorder (e.g., epilepsy, dementia); (c) missing or incomplete data on overall diet quality or impulsivity; and (d) missing genetic data or non-European ancestry. The final sample of N = 33,047 participants was included in the analyses (see flowchart, Figure 1). 

### 2.2. Outcome: Impulsivity

At baseline, 32 selected items from the 240-item NEO personality index (The NEO personality inventory–revised (NEO-PI-R) is a personality inventory that assesses an individual on five personality dimensions [23]) were administered, including all items constituting of the impulsivity facet of the neuroticism scale, the excitement-seeking facet of the extraversion scale, and the deliberateness and self-discipline facets of the conscientiousness scale. Each facet consists of eight items scored on a five-point Likert scale (e.g., “I often do things without thinking”; 1 = “strongly disagree” to 5 = “strongly agree”), resulting in facet sum scores ranging from 8 to 40. The self-discipline and deliberateness facet sum scores were inverted, such that higher scores indicated more impulsive behaviors. Next, a single principal component was extracted based on Pearson’s correlations between the four facet sum scores. All sum scores loaded positively on the principal component (deliberateness [inverted]: 0.822; impulsivity: 0.816; self-discipline [inverted]: 0.678; excitement-seeking: 0.371) indicated a unitary construct. The construct was named the trait impulsivity and a single impulsivity score per subject was derived. Note that the calculation of the principal component scores was based on N = 109,543 (i.e., on all eligible participants with and without genetic data) to obtain more precise estimations.

### 2.3. Predictor: ADHD PRS

For some of the Lifelines participants, genome-wide genotype data were available. Among participants in the current study, DNA samples were genotyped using the Illumina Global Screening array (N = 27,075) and Illumina CytoSNP12v2 array (N = 5972). Following quality control, both genotyping datasets were then imputed into the Sanger imputation server using the Haplotype Reference Consortium panel r1.1 [24]. Details of genotyping, quality control, and imputation in Lifelines have been published elsewhere [25]. 

The ADHD PRS was calculated to represent the cumulative effects of many common genetic variants on ADHD. Because the GWASs on ADHD [8,9] were more powerful and identified more loci than the GWAS on impulsive personality traits [7], we built the ADHD PRS using the most recent meta-GWAS on ADHD conducted on 38,691 ADHD cases and 186,843 controls with European ancestry [9]. Multiallelic SNPs and SNPs with ambiguous strands (A/T or C/G) were removed from the ADHD GWAS summary results. Overlapping SNPs across GWAS results and the Lifelines sample with minor allele frequency (MAF > 1%) and imputation quality (INFO > 0.8) were kept. To obtain an independent set of SNPs, an LD-driven clumping procedure was performed in PLINK (r2 < 0.1, 250 kb window) using the LD reference panel of 503 European samples from phase 3 of the 1000 Genomes Project [26]. For each individual, the PRSs were calculated by multiplying the risk allele dosages for each SNP by its respective weight (the log of the odds ratio) and then summing all SNPs in the score. Scores were constructed at 11 selected *p*-value thresholds (5 × 10^−8^, 1 × 10^−7^, 1 × 10^−6^, 1 × 10^−5^, 1 × 10^−4^, 1 × 10^−3^, 0.01, 0.05, 0.1, 0.5, 1) and were then standardized using z-score transformations in R software. Finally, a principal component analysis (PCA) was performed on these scores and the first principal component was extracted as the final ADHD PRS. This approach is called the PRS-PCA approach, that avoids optimizing the parameters to construct the PRS, and has been shown to be an unbiased and powerful way to index polygenic risk [27]. 

To calculate the percentage of impulsivity variance explained by the ADHD PRS, we generated the residuals from a linear regression of impulsivity against age, sex, genotyping chip, four genetic principal components (PCs), and interactions between genotyping chip and each genetic PC, then we fit a second linear model for the residuals with the ADHD PRS. The adjusted R2 of the second model is the estimation of the percentage of the impulsivity variance explained by the ADHD PRS. The ADHD PRS explained 0.39% (*p* = 5.4 × 10^−30^) of the variance on impulsivity.

### 2.4. Moderator: Diet

Participants’ past-month food intake was collected using a 110-item semi-quantitative food frequency questionnaire (FFQ) [28]. For each food-item, participants reported consumption frequency on a seven-point categorical scale (ranging from “not this month” to “6–7 days/week”). Portion sizes were estimated using natural portions and commonly used household measures. From the FFQ data, total energy intake in kcal/day was estimated using the 2011 Dutch food composition database [29]. Reliability was assessed by comparing the total daily energy intake to the basal metabolic rate (BMR), as estimated by the Schofield equations [30]. Participants reporting <0.79 or >2.49 times the amount of energy required according to age, sex, and height (i.e., plus/minus 2SD) were deemed unreliable and excluded.

Four indices of poor diet were computed: low overall diet quality, excess intake of energy, excess intake of fat, and excess intake of free sugars. The lifelines diet score (LLDS) [28] is a food-based assessment of the overall diet quality, based on international evidence for diet-disease relations and in line with the 2015 Dutch dietary guidelines [31]. Intake of nine food groups with established positive health effects (vegetables, fruits, whole-grain products, legumes and nuts, fish, oils and soft margarines, unsweetened dairy, coffee, and tea) in grams per 1000 kcal is categorized into quintiles and scored 0–4 points. Inversely, the intake of three food groups with negative health effects (red/processed meat, butter and hard margarines, and sugar-sweetened beverages) is scored as 4–0 points. Food groups for which evidence of health effects is absent/weak are not taken into account. The LLDS is calculated as the sum of positive and negative food group quintile scores (range 0–48). Higher scores indicate a healthier diet. For the current study, we inverted the LLDS score (LLDS-I), such that higher scores represented poor overall diet quality. 

Excessive intake of energy, fat, and free sugars were specified to reflect adherence to general health recommendations. Energy intake ratio (KCAL) was defined as the daily total energy intake relative to the BMR and scaled, such that KCAL = 1 indicates perfect adherence and KCAL > 1 indicates excessive intake. Since energy deficits (KCAL < 1) are not generally linked to either healthy or unhealthy dietary habits, KCAL < 1 was set to 1. Free sugar intake ratio (SUGAR) was defined as the percentage of the total daily energy intake provided by free sugars (i.e., natural sugars and added sugars) relative to the WHO-recommended maximum daily intake of 10% [32]. The fat intake ratio (FAT) was defined as the percentage of total daily energy intake provided by fat relative to the WHO-recommended maximum daily intake of 30% [32]. The WHO guidelines specify maximum rather than optimal intake. As the optimal intake of free sugars and fat is unknown, health effects of below-maximum intake might in theory be beneficial, neutral, or detrimental. Excessive intake, by contrast, is known to have detrimental health effects. We thus argue that below-maximum and excessive intake should not be placed on a continuum, and replaced SUGAR < 1 and FAT < 1 by 1, indicating non-excessive intake. The four indicators of an unhealthy diet were modestly correlated (Supplementary Materials Table S1). 

To aid in the interpretation and visualization of interactions between genetic risk and unhealthy dietary behaviors, participants were grouped into tertiles for overall diet quality (Q1: LLDS-I = 1–21; Q2: LLDS-I = 21–26; Q3: LLDS-I > 26), intake of energy (Q1: KCAL = 1–1.14; Q2: KCAL = 1.14–1.41; Q3: KCAL > 1.41), and intake of fat (Q1: FAT = 1–1.12; Q2: FAT = 1.12–1.25; Q3: FAT > 1.25). A binary grouping was made for the intake of sugar, as >33% of participants reported a non-excessive intake (Q1: SUGAR = 1; Q2: SUGAR > 1). 

### 2.5. Secondary Moderators: Other Lifestyle Variables

Physical activity was assessed using the SQUASH questionnaire, in which participants self-reported the frequency, duration, and intensity of their habitual activities in a regular week [33]. Based on their report, we calculated the minutes per week spent in moderate-to-vigorous physical activity (MVPA) during commuting and leisure time, including sports [34]. The distribution of MVPA was positively skewed. We thus rescaled MVPA into semi-continuous quintile scores (1 = 0 to 60 min per week; 2 = 60–150 min/week; 3 = 150–255 min/week; 4 = 250–420 min/week; 5 = >420 min/week).

Sleep duration in hours and minutes was assessed in a single questionnaire item. Both short and long sleep are associated with poor (mental) health outcomes, indicating non-linear associations. Moreover, sleep duration changes with age in a sex-dependent manner [35]. We therefore defined short and long sleep duration as belonging to the lowest and highest decile of sleep duration residuals regressed on age and sex, respectively, and defined all other participants as the reference group.

Alcohol intake was derived from the FFQ. Participants reported the frequency and quantity of their intake of beer, red/rose wine, white wine, fortified wine (e.g., sherry, port), liquor/distilled alcoholic drinks (e.g., rum, whiskey), and other alcoholic drinks. Items were weighted by their alcohol content and summed across drink types to derive alcohol intake in grams per day. Next, the drinking level was categorized as abstinent, occasional (<2.5 g/day), light (2.5–14.9 g/day), moderate (15–29.9 g/day), or heavy (>30 g/day) [36]. The abstinent group was set as the reference group.

Participants were classified as current smokers if they reported having smoked cigarettes, cigars, cigarillos, or a pipe on one or more occasions within one month prior to the baseline assessment. Participants were classified as past smokers if they reported having smoked cigarettes, cigars, cigarillos, or a pipe for one year or longer but not during the past month. All others were classified as never-smokers (reference group).

### 2.6. Covariates

Recent work by Akimova et al. [37] indicates that the presence of gene-environment correlation (rGE) [38] (e.g., between the ADHD PRS and diet [39]) may yield biased results of mostly the main effects in the presence of unobserved confounders. Thus, we included a comprehensive set of covariates in the models in the main analyses (e.g., four socioeconomic status (SES) indices as major potential confounders of the relation between diet/other lifestyles, and impulsivity). Covariates were chosen based on the results from previous studies [40], including: demographic: age, sex, body mass index (BMI) in kg/m^2^; SES: neighborhood socioeconomic status, which was calculated by combining the postal code of participants and the status score of that area, disposable household income, which was defined as the monthly household income adjusted for household size using equivalence factors from the Statistics Netherlands (CBS) website [41], educational attainment (low/middle/high), and occupational status using the occupational prestige scale based on the International Standard Classification of Occupations (ISCO-08) [42]. For participants aged < 25, all socioeconomic parameters except neighborhood SES were based on parental data; non-communicable diseases: lifetime diagnosis (yes/no) of cardiovascular disease, cancer, diabetes, or liver cirrhosis; mental illness: current diagnosis of depression (yes/no) or anxiety disorder (yes/no); and stress: past year number of stressful life events and long-term difficulties. Missing data points for covariates were imputed using multivariate imputation by chained equations (MICEs [43]), applying classification tree predictions for categorical variables and regression tree predictions for continuous variables. Categorical values were derived after the imputation of the underlying continuous variables.

### 2.7. Statistical Analyses

Models were estimated in multiple steps. Step 1: in the basic linear model, impulsivity was predicted from the ADHD PRS plus all covariates (age, sex, BMI, SES, lifetime diagnosis of non-communicable diseases, current diagnosis of depression or anxiety disorder, and past year number of stressful life events and long-term difficulties). Step 2a: In four separate models, each unhealthy diet indicator and its interaction with the ADHD PRS was added to the basic model. This step evaluated whether an unhealthy diet, as indicated by poor diet quality (LLDS-I), higher intake of fat (FAT), higher intake of sugar (SUGAR), or higher intake of energy (KCAL) might modify the genetic risk of impulsive behaviors. Step 2b: Exploratively, in four separate models, the other lifestyle predictors (smoking, drinking, sleep duration, and physical activity) and their interactions with the ADHD PRS were added to the basic model. In all steps, we also adjusted for genotyping chip, four genetic principal components (PCs) accounting for population stratification, and interactions between genotyping chip and each genetic PC.

All analyses were performed in R version 3.5.2. The outcome impulsivity score was normally distributed. *p* value < 0.0125 Bonferroni-adjusted for multiple testing (four models in step 2a and step 2b) was considered statistically significant.

### 2.8. Sensitivity Analyses

Further simulation analyses by Akimova et al. [37] revealed that interactions between unobserved confounders and environmental exposures may inflate the effect of gene-by-environment interaction when not taken into account. Therefore, in the sensitivity analyses, we also included the interactions between each diet/other lifestyles and all four SES indices to test whether and to what extent these diet/other lifestyles × SES interactions had inflated the effect of ADHD PRS × diet/other lifestyles on impulsivity. In addition, we calculated Pearson’s correlations between the ADHD PRS and diet/other lifestyles as a measure of the presence of rGE.

## 3. Results

### 3.1. Participants’ Characteristics

Table 1 presents the characteristics of the study participants. A total of 33,047 participants were included in the analyses, among whom 19,767 (59.8%) were females. The mean age was 42.12 years.

LLDS-I represents the inverted lifelines diet score, a higher LLDS-I means a poor overall diet quality; KCAL, energy intake ratio, defined as the daily total energy intake relative to the BMR; FAT, the fat intake ratio is defined as the percentage of the total daily energy intake provided by fat relative to the WHO-recommended maximum daily intake of 30%; SUGAR, the free sugar intake ratio, defined as the percentage of total daily energy intake provided by free sugars (i.e., natural sugars and added sugars) relative to the WHO-recommended maximum daily intake of 10%. MVPA indicates minutes per week spent on moderate-to-vigorous physical activity.

### 3.2. G: ADHD PRS and Impulsivity

The ADHD PRS was statistically significantly associated with impulsivity in the general population (B = 0.03 (95% CI: 0.02, 0.04); *p* = 2.61 × 10^−9^) (Table 2). Additionally, a younger age, being female, a higher BMI, a lower education attainment, a lower occupational status, a larger past year number of stressful life events and life-term difficulties, and a current diagnosis of depression and anxiety were associated with higher impulsivity (Table 2). 

### 3.3. GxE: Moderation by Diet Indicators

**Figure 2:**
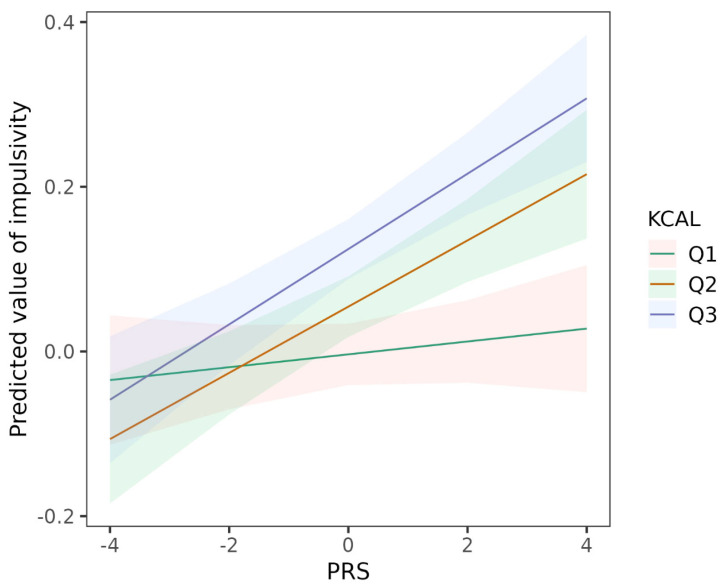
Mean predicted value of impulsivity with the ADHD PRS for individuals at each tertile of energy intake. KCAL represents the intake of energy. Q1 indicates tertile 1; Q2, tertile 2; Q3, tertile 3.

Table 3 shows the results of diet indicators and their interactions with the ADHD PRS on impulsivity. Poorer overall diet quality (e.g., highest vs. lowest tertile, B = 0.14 (95% CI: 0.115, 0.166) when the ADHD PRS equals 0 (the average), *p* = 6.91 × 10^−27^), higher energy intake (e.g., highest vs. lowest tertile, B = 0.128 (95% CI: 0.103, 0.153) when the ADHD PRS equals 0, *p* = 2.81 × 10^−23^), and higher intake of fat (e.g., highest vs. lowest tertile, B = 0.145 (95% CI: 0.12, 0.169) when the ADHD PRS equals to 0, *p* = 9.50 × 10^−32^) were significantly associated with higher impulsivity and showed dose-response relationships. No association was detected between the intake of sugar and impulsivity. For the possible interaction effects, only energy intake was found to moderate the association between ADHD PRS and impulsivity. Specifically, a high intake of energy amplified the association between ADHD PRS and impulsivity (e.g., for the interaction term of ADHD PRS and highest tertile of intake of energy, B = 0.038 (95% CI: 0.014, 0.062); *p* = 0.002), as shown by the stronger genetic effects in participants with the higher energy intake (Table 3, Figure 2). 

ADHD PRS × variable represents the interaction term between ADHD PRS and the variable. LLDS-I represents the inverted lifelines diet score, a higher LLDS-I means poor overall diet quality; KCAL, energy intake ratio; FAT, fat intake ratio; SUGAR, free sugar intake ratio. Ref, reference group.

### 3.4. Exploration: Other Lifestyles

Table 4 shows the results of other lifestyle factors and their interactions with the ADHD PRS on impulsivity. Short and long sleep duration, current and past smoking, higher alcohol intake, and more time spent on MVPA were associated with higher impulsivity, but no interaction effect was observed between these lifestyles and the ADHD PRS on impulsivity. 

MVPAQ indicates semi-continuous quintile scores of minutes per week spent in moderate-to-vigorous physical activity. Ref, reference group.

### 3.5. Sensitivity Analyses

Small but significant rGEs were present between the ADHD PRS and all four diet indicators, sleep hours, and smoking (Supplementary Materials Table S2). Adjusting for diet/other lifestyles × SES slightly attenuated the effect size of the ADHD PRS × intake of energy interaction but the interaction remained significant (Supplementary Materials Table S3).

## 4. Discussion

In this study, we investigated whether an unhealthy diet and other lifestyle behaviors may modify the genetic risk of impulsivity in 33,047 participants from the Dutch Lifelines cohort. The results showed that a high intake of energy amplified the association between the polygenic load of ADHD and impulsivity, as shown by stronger genetic effects on impulsivity within participants with the higher energy intake. No other diet or lifestyle behaviors were found to statistically significantly modify the ADHD PRS-impulsivity association.

Consistent with the literature [11,12], we showed that ADHD PRS was positively associated with trait impulsivity. The findings showed the utility of ADHD PRS on explaining impulsivity traits in the general population, and also corroborated the positive genetic correlation between ADHD and measures of impulsivity (e.g., rg = 0.43 between ADHD and lack of premeditation [7]). However, it is worth noting that the ADHD PRS was found to explain only a small proportion of variance in trait impulsivity, as in previous studies (e.g., Martin et al. found that polygenic risk ADHD explained 0.2% of variance in hyperactive-impulsive traits) [11,12]. Larger GWASs for ADHD or other impulsivity-related traits and improved approaches to calculating genetic predictors (e.g., MTAG [44], GenomicSEM [45]) promise to enhance the genetic prediction of impulsivity in the future. 

In addition to the genetic factors, we demonstrated that diet (i.e., poorer overall diet quality, higher intake of energy, and higher intake of fat) and other lifestyle factors (i.e., short and long sleep duration, current and past smoking, higher alcohol intake, and more time spent on MVPA) were associated with a higher level of impulsivity. The standardized effect sizes of the diet indicators roughly ranged from 0.03 to 0.07, which is similar to the effect sizes for ADHD PRS, educational attainment, occupational status, past year number of stressful life events, and current depression/anxiety, but much weaker compared to the effects of age, BMI, and past year number of life-term difficulties. Earlier studies observed similar associations for diet [14], smoking [46], and drinking [47]. Impulsivity has been shown to be negatively associated with sleep duration [48], but our study found that both short and long sleep duration were associated with higher impulsivity, suggesting the relationship between sleep duration and impulsivity may not be linear. Interestingly, we found that time spent on MVPA was positively associated with impulsivity. This is in line with several previous studies [49,50]. For instance, a longitudinal study on Swedish children showed that hyperactivity/impulsivity symptoms in childhood were associated with a higher likelihood of being physically active in adolescence, whereas the opposite was true for inattention [49]. As the cross-sectional and observational nature of our study prevents causal inferences, these observed associations might indicate an effect of impulsivity on diet and other lifestyle factors, an effect of diet and other lifestyle factors on impulsivity, or both. Future longitudinal studies and causal inference methods, such as Mendelian randomization studies, may help to clarify the directionality of these associations. 

Our findings highlight the identification of the gene-diet interaction on trait impulsivity. We found that a high intake of energy amplified the association between ADHD PRS and impulsivity. This is in line with an earlier twin study showing that genetic influences on ADHD symptoms increased at higher levels of unhealthy food intake [18] It also empirically supports the diathesis–stress model [51] of gene-environment interaction in which genes and stressful environments exert risks synergistically (with the stressors being a high intake of energy). We explored four diet indicators and other lifestyle factors, and observed a significant interaction between the intake of energy and ADHD PRS on trait impulsivity, which emphasized the critical role of energy intake in moderating genetic effects on impulsivity. An alternative explanation may be that the FFQ may measure the energy intake more accurately than other diet indicators [52,53], so it is easier to detect the interaction between the intake of energy and ADHD PRS on trait impulsivity. Potentially, individuals with a genetic predisposition to ADHD may benefit from reducing excessive energy intake in their daily diet. 

Research on the emerging field of nutrigenomics [54,55] may shed light on understanding how dietary factors moderate the genetic effects on impulsivity. From a nutrigenomics perspective, nutrients are dietary signals that can be detected by the cellular sensor systems and then influence gene expression, leading to changes in protein and metabolite production. Notably, nutrients can produce long-lasting epigenetic effects that regulate gene expression without altering the DNA sequence, but through DNA methylation, histone modification, and chromatin-associated proteins [56]. For example, animal studies have shown that high-fat or high-energy diets were associated with increased DNA methylation and decreased expression of genes critical for dopamine receptors in brain regions related to reward and motivated behaviors [57,58]. Future molecular studies integrating various omics data may yield more insights into the underlying mechanisms of diet-gene interaction on impulsivity. 

We observed significant but very small rGEs between the ADHD PRS and all four diet indicators, sleep hours, and smoking. Although the existence of rGE may inflate the results of GxE interaction in the presence an interaction between unobserved confounders and environmental exposures [37], our sensitivity analyses showed that ADHD PRS × intake of energy interaction remained significant after adjusting for diet/other lifestyles × SES. It indicates the robustness of the interaction between ADHD PRS and intake of energy on impulsivity. 

To our knowledge, we are the first to detect diet-gene interactions on impulsivity in a large sample of adults using a polygenic score approach and the most recent GWAS of ADHD. In addition, we explored a broad range of diet scores/indices and lifestyles with adjustments of a comprehensive set of covariates.

The study has several limitations. First, self-report measures on diet and other lifestyle factors were subject to reporting and recall bias. Furthermore, diet was only assessed, once which could not capture changes over time. Second, ADHD PRS only explained a small proportion of variance in trait impulsivity, limiting the power to detect gene-environment interactions. Finally, we used meta-GWAS results of European ancestry and applied PRS to the same ancestry, so further studies may be required to determine whether our results can be generalized to other non-European ancestries. 

## 5. Conclusions

The present study showed that a high intake of energy exacerbated the genetic risk of impulsivity. These findings support the diathesis–stress model, providing insights into the role of diet and genetic factors on impulsivity. Future molecular studies are required to better comprehend the underlying mechanisms of these diet-gene interactions.

## Figures and Tables

**Table 1:** The characteristics ^a^ of the study participants.

	Overall	Male	Female
N	33,047	13,280	19,767
Age (years)	42.12 (12.35)	42.40 (12.22)	41.93 (12.43)
Impulsivity score ^b^	0.01 (0.99)	−0.04 (0.98)	0.04 (0.99)
ADHD PRS ^c^	−0.01 (1.00)	−0.02 (0.99)	0.00 (1.00)
**Diet**			
Overall diet quality (LLDS_I)	23.22 (5.99)	24.73 (5.57)	22.20 (6.04)
Intake of energy (KCAL)	1.28 [1.08, 1.50]	1.28 [1.08, 1.51]	1.27 [1.08, 1.49]
Intake of sugar (SUGAR)	1.06 [1.00, 1.43]	1.10 [1.00, 1.48]	1.04 [1.00, 1.39]
Intake of fat (FAT)	1.18 [1.08, 1.28]	1.19 [1.08, 1.29]	1.18 [1.07, 1.28]
**Lifestyles**			
MVPA (minutes per week)	200.00 [75.00, 370.00]	210.00 [60.00, 395.00]	195.00 [90.00, 360.00]
Sleep duration (hours)	7.49 (0.83)	7.32 (0.79)	7.60 (0.84)
Sleep duration group (*n*, %)			
Normal	26,355 (80.0)	10,636 (80.4)	15,719 (79.8)
Short	3,283 (10.0)	1,374 (10.4)	1909 (9.7)
Long	3,289 (10.0)	1215 (9.2)	2074 (10.5)
Alcohol intake (grams)	4.73 [1.24, 10.80]	7.96 [2.92, 15.81]	2.70 [0.63, 6.92]
Alcohol intake group (*n*, %)			
Abstinent	5186 (15.7)	1000 (7.5)	4186 (21.2)
Occasional	6809 (20.6)	1836 (13.8)	4973 (25.2)
Light	15,672 (47.4)	6871 (51.7)	8801 (44.5)
Moderate	4462 (13.5)	2800 (21.1)	1662 (8.4)
Heavy	918 (2.8)	773 (5.8)	145 (0.7)
Smoke (*n*, %)			
Current	6329 (19.3)	2827 (21.5)	3502 (17.9)
Never	16,071 (49.1)	6277 (47.7)	9794 (50.0)
Past	10,330 (31.6)	4044 (30.8)	6286 (32.1)
**Covariates**			
Lifetime diagnosis of non-communicable diseases (*n*, %)	10,434 (31.6)	4051 (30.5)	6383 (32.3)
Current diagnosis of depression (*n*, %)	835 (2.5)	239 (1.8)	596 (3.0)
Current diagnosis of anxiety (*n*, %)	2354 (7.1)	675 (5.1)	1679 (8.5)
Past year number of stressful life events	1.00 [0.00, 2.00]	1.00 [0.00, 2.00]	1.00 [0.00, 2.00]
Past year number of life-term difficulties	2.00 [1.00, 4.00]	2.00 [1.00, 3.00]	2.00 [1.00, 4.00]
BMI (kg/m^2^)	25.51 (4.04)	25.91 (3.49)	25.24 (4.34)
Educational attainment (*n*, %)			
Low	8362 (25.5)	3349 (25.4)	5013 (25.6)
Middle	13,374 (40.8)	5139 (38.9)	8235 (42.0)
High	11,068 (33.7)	4708 (35.7)	6360 (32.4)
Occupational status	43.88 (13.08)	45.41 (12.47)	42.85 (13.37)
Neighbourhood socioeconomic status	−0.61 (1.08)	−0.59 (1.08)	−0.62 (1.08)
Disposable household income (EUR)	1641.75 (517.12)	1689.11 (507.71)	1608.97 (521.04)

^a^ Descriptives are either the mean (SD) or median [interquartile range], depending on the distribution of the variable or number (%), as specified in the table. ^b^ Impulsivity score was calculated as a single principal component, and a higher score represents a higher level of impulsivity. ^c^ The ADHD PRS was standardized.

**Table 2:** Results of the associations between the ADHD PRS and covariates with impulsivity.

	B (95% CI)	*p* Value	Beta
ADHD PRS	0.03 (0.02, 0.04)	2.61 × 10^−9^	0.031
Age (years)	−0.021 (−0.022, −0.02)	0	−0.258
Sex = female	0.036 (0.016, 0.057)	4.71 × 10^−4^	0.018
BMI (kg/m^2^)	0.03 (0.028, 0.033)	5.68 × 10^−116^	0.123
Neighbourhood socioeconomic status	−0.011 (−0.021, −0.002)	0.019	−0.012
Education attainment (ref = low)			
Middle	−0.086 (−0.112, −0.06)	1.50 × 10^−10^	−0.043
High	−0.185 (−0.217, −0.153)	1.29 × 10^−29^	−0.088
Disposable household income (euros)	2.25×10^−5^ (1.16 × 10^−6^, 4.39 × 10^−5^)	0.039	0.011
Occupational status	−0.002 (−0.003, −0.001)	2.37 × 10^−6^	−0.029
Lifetime diagnosis of non-communicable diseases	−0.016 (−0.039, 0.007)	0.181	−0.007
Past year number of stressful life events	0.021 (0.012, 0.029)	6.96 × 10^−7^	0.027
Past year number of life-term difficulties	0.091 (0.086, 0.096)	2.61 × 10^−292^	0.215
Current diagnosis of depression	0.259 (0.192, 0.326)	3.83 × 10^−14^	0.041
Current diagnosis of anxiety	0.156 (0.115, 0.197)	1.13 × 10^−13^	0.040

**Table 3:** Results ^a^ of the diet indicators and their interactions with the ADHD PRS on impulsivity.

	B (95% CI)	*p* Value	Beta
**Overall diet quality (LLDS_I) (ref = Q1) ^b^**			
LLDS_I_Q2	0.058 (0.033, 0.083)	5.36 × 10^−6^	0.027
LLDS_I_Q3	0.14 (0.115, 0.166)	6.91 × 10^−27^	0.068
ADHD PRS	0.025 (0.008, 0.043)	0.004	0.025
ADHD PRS × LLDS_I_Q2	0.009 (−0.016, 0.034)	0.467	0.005
ADHD PRS × LLDS_I_Q3	0.005 (−0.019, 0.029)	0.674	0.003
**Intake of energy (KCAL) (ref = Q1) ^b^**			
KCAL_Q2	0.058 (0.034, 0.082)	3.08 × 10^−6^	0.028
KCAL_Q3	0.128 (0.103, 0.153)	2.81 × 10^−23^	0.061
ADHD PRS	0.008 (−0.009, 0.025)	0.371	0.008
ADHD PRS × KCAL_Q2	0.032 (0.008, 0.057)	0.009	0.019
ADHD PRS × KCAL_Q3	0.038 (0.014, 0.062)	0.002	0.022
**Intake of fat (FAT) (ref = Q1) ^b^**			
FAT_Q2	0.065 (0.041, 0.089)	1.32 × 10^−7^	0.031
FAT_Q3	0.145 (0.12, 0.169)	9.50 × 10^−32^	0.069
ADHD PRS	0.019 (0.002, 0.036)	0.030	0.019
ADHD PRS × FAT_Q2	0.017 (−0.007, 0.041)	0.161	0.010
ADHD PRS × FAT_Q3	0.016 (−0.008, 0.04)	0.192	0.009
**Intake of sugar (SUGAR) (ref = Q1) ^c^**			
SUGAR_Q2	0.001 (−0.02, 0.021)	0.940	0.0004
ADHD PRS	0.031 (0.018, 0.045)	4.42 × 10^−6^	0.032
ADHD PRS × SUGAR_Q2	−0.002 (−0.022, 0.018)	0.833	−0.001

^a^ Results from the models adjusted for all covariates. ^b^ Q1 indicates tertile 1; Q2, tertile 2; Q3, tertile 3. ^c^ Q1: free sugar intake ratio (SUGAR) ≤ 1; Q2: SUGAR > 1. Ref, reference group.

**Table 4:** Results ^a^ of other lifestyles and their interactions with the ADHD PRS on impulsivity.

	B (95% CI)	*p* Value	Beta
**Sleep duration** **(ref = middle sleep duration)**			
Short sleep duration	0.078 (0.045, 0.112)	4.19 × 10^−6^	0.024
Long sleep duration	0.049 (0.016, 0.082)	0.004	0.015
ADHD PRS	0.027 (0.015, 0.038)	3.10 × 10^−6^	0.027
ADHD PRS × short sleep duration	0.022 (−0.011, 0.055)	0.187	0.007
ADHD PRS × long sleep duration	0.011 (−0.022, 0.044)	0.508	0.004
**Smoking (ref = never smoking)**			
Current smoking	0.302 (0.275, 0.328)	6.70 × 10^−107^	0.120
Past smoking	0.208 (0.185, 0.232)	7.50 × 10^−67^	0.098
ADHD PRS	0.03 (0.016, 0.044)	3.55 × 10^−5^	0.030
ADHD PRS × current smoking	−0.012 (−0.038, 0.015)	0.382	−0.005
ADHD PRS × past smoking	−0.014 (−0.036, 0.009)	0.227	−0.008
**Alcohol intake** **(ref = no alcohol intake)**			
Occasional alcohol intake	0.091 (0.058, 0.124)	5.37 × 10^−8^	0.037
Light alcohol intake	0.267 (0.238, 0.296)	1.06 × 10^−71^	0.135
Moderate alcohol intake	0.435 (0.397, 0.472)	4.44 × 10^−113^	0.150
Heavy alscohol intake	0.498 (0.433, 0.563)	6.13 × 10^−51^	0.083
ADHD PRS	0.025 (0.001, 0.049)	0.045	0.025
ADHD PRS × occasional alcohol intake	0.018 (−0.015, 0.05)	0.287	0.008
ADHD PRS × light alcohol intake	−0.001 (−0.029, 0.028)	0.965	0.000
ADHD PRS × moderate alcohol intake	0.001 (−0.035, 0.037)	0.944	0.000
ADHD PRS × heavy alcohol intake	0.042 (−0.022, 0.105)	0.196	0.007
**Physical activity**			
MVPAQ	0.019 (0.012, 0.026)	3.87 × 10^−7^	0.027
ADHD PRS	0.03 (0.006, 0.054)	0.016	0.030
ADHD PRS × MVPAQ	−0.001 (−0.008, 0.007)	0.880	−0.002

^a^ Results from the models adjusted for all covariates.

## Data Availability

Lifelines is a facility that is open for all researchers; information on application and data access procedures is available at https://www.lifelines.nl/researcher/how-to-apply (accessed on 23 February 2023).

## Supplementary Materials

The following supporting information can be downloaded at: https://www.mdpi.com/article/10.3390/nu15071625/s1, Table S1: Pearson’s correlations between the four indicators of dietary habits; Table S2: Gene-environment correlations between the PRS and diet/other lifestyles; Table S3: Sensitivity analyses results of energy intake and its interaction with the PRS after including the interactions between energy intake and all four SES indices.

## Appendix A. Lifelines Cohort Study-UGLI Group Authors

Raul Aguirre-Gamboa ^1^, Patrick Deelen ^1^, Lude Franke ^1^, Jan A. Kuivenhoven ^2^, Esteban A. Lopera Maya ^1^, Ilja M. Nolte ^3^, Serena Sanna ^1^, Harold Snieder ^3^, Morris A. Swertz ^1^, Peter M. Visscher ^3,4^, Judith M. Vonk ^3^ and Cisca Wijmenga ^1^

1. Department of Genetics, University Medical Center Groningen, University of Groningen, 9700 Groningen, The Netherlands
2. Department of Pediatrics, University Medical Center Groningen, University of Groningen, 9700 Groningen, The Netherlands
3. Department of Epidemiology, University Medical Center Groningen, University of Groningen, 9700 Groningen, The Netherlands
4. Institute for Molecular Bioscience, The University of Queensland, Brisbane, QLD 4072, Australia

## References

[1] Moeller F.G., Barratt E.S., Dougherty D.M., Schmitz J.M., Swann A.C. (2001). Psychiatric aspects of impulsivity. Am. J. Psychiatry.

[2] Potenza M.N. (2007). To do or not to do? The complexities of addiction, motivation, self-control, and impulsivity. Am. J. Psychiatry.

[3] Chamorro J., Bernardi S., Potenza M.N., Grant J.E., Marsh R., Wang S., Blanco C. (2012). Impulsivity in the general population: A national study. J. Psychiatr. Res..

[4] Faraone S.V., Asherson P., Banaschewski T., Biederman J., Buitelaar J.K., Ramos-Quiroga J.A., Rohde L.A., Sonuga-Barke E.J.S., Tannock R., Franke B. (2015). Attention-deficit/hyperactivity disorder. Nat. Rev. Dis. Prim..

[5] Sciberras E., Streatfeild J., Ceccato T., Pezzullo L., Scott J.G., Middeldorp C.M., Hutchins P., Paterson R., Bellgrove M.A., Coghill D. (2022). Social and Economic Costs of Attention-Deficit/Hyperactivity Disorder Across the Lifespan. J. Atten. Disord..

[6] Bezdjian S., Baker L.A., Tuvblad C. (2011). Genetic and environmental influences on impulsivity: A meta-analysis of twin, family and adoption studies. Clin. Psychol. Rev..

[7] Sanchez-Roige S., Fontanillas P., Elson S.L., Gray J.C., de Wit H., MacKillop J., Palmer A.A. (2019). Genome-Wide Association Studies of Impulsive Personality Traits (BIS-11 and UPPS-P) and Drug Experimentation in up to 22,861 Adult Research Participants Identify Loci in the CACNA1I and CADM2 genes. J. Neurosci..

[8] Demontis D., Walters R.K., Martin J., Mattheisen M., Als T.D., Agerbo E., Baldursson G., Belliveau R., Bybjerg-Grauholm J., Bækvad-Hansen M. (2019). Discovery of the first genome-wide significant risk loci for attention deficit/hyperactivity disorder. Nat. Genet..

[9] Demontis D., Walters G.B., Athanasiadis G., Walters R., Therrien K., Nielsen T.T., Farajzadeh L., Voloudakis G., Bendl J., Zeng B. (2023). Genome-wide analyses of ADHD identify 27 risk loci, refine the genetic architecture and implicate several cognitive domains. Nat. Genet..

[10] Larsson H., Anckarsater H., Råstam M., Chang Z., Lichtenstein P. (2012). Childhood attention-deficit hyperactivity disorder as an extreme of a continuous trait: A quantitative genetic study of 8,500 twin pairs. J. Child Psychol. Psychiatry.

[11] Martin J., Hamshere M.L., Stergiakouli E., O’Donovan M.C., Thapar A. (2014). Genetic risk for attention-deficit/hyperactivity disorder contributes to neurodevelopmental traits in the general population. Biol. Psychiatry.

[12] Agnew-Blais J.C., Belsky D.W., Caspi A., Danese A., Moffitt T.E., Polanczyk G.V., Sugden K., Wertz J., Williams B.S., Lewis C.M. (2021). Polygenic Risk and the Course of Attention-Deficit/Hyperactivity Disorder from Childhood to Young Adulthood: Findings From a Nationally Representative Cohort. J. Am. Acad. Child Adolesc. Psychiatry.

[13] Stergiakouli E., Hamshere M., Holmans P., Langley K., Zaharieva I., Hawi Z., Kent L., Gill M., Williams N., Owen M.J. (2012). Investigating the contribution of common genetic variants to the risk and pathogenesis of ADHD. Am. J. Psychiatry.

[14] Bénard M., Bellisle F., Kesse-Guyot E., Julia C., Andreeva V.A., Etilé F., Reach G., Dechelotte P., Tavolacci M.-P., Hercberg S. (2019). Impulsivity is associated with food intake, snacking, and eating disorders in a general population. Am. J. Clin. Nutr..

[15] Wiles N.J., Northstone K., Emmett P., Lewis G. (2009). “Junk food” diet and childhood behavioural problems: Results from the ALSPAC cohort. Eur. J. Clin. Nutr..

[16] Loewen O.K., Maximova K., Ekwaru J.P., Ohinmaa A., Veugelers P.J. (2020). Adherence to Life-Style Recommendations and Attention-Deficit/Hyperactivity Disorder: A Population-Based Study of Children Aged 10 to 11 Years. Psychosom. Med..

[17] Assary E., Vincent J.P., Keers R., Pluess M. (2018). Gene-environment interaction and psychiatric disorders: Review and future directions. Semin. Cell Dev. Biol..

[18] Li L., Taylor M.J., Bälter K., Xie T., Solberg B.S., Haavik J., Vásquez A.A., Hartman C.A., Larsson H. (2021). Gene-Environment Interactions in Attention-Deficit/Hyperactivity Disorder Symptom Dimensions: The Role of Unhealthy Food Habits. Genes.

[19] Kahn R.S., Khoury J., Nichols W.C., Lanphear B.P. (2003). Role of dopamine transporter genotype and maternal prenatal smoking in childhood hyperactive-impulsive, inattentive, and oppositional behaviors. J. Pediatr..

[20] Brookes K.-J., Mill J., Guindalini C., Curran S., Xu X., Knight J., Chen C.-K., Huang Y.-S., Sethna V., Taylor E. (2006). A common haplotype of the dopamine transporter gene associated with attention-deficit/hyperactivity disorder and interacting with maternal use of alcohol during pregnancy. Arch. Gen. Psychiatry.

[21] Palladino V.S., McNeill R., Reif A., Kittel-Schneider S. (2019). Genetic risk factors and gene-environment interactions in adult and childhood attention-deficit/hyperactivity disorder. Psychiatr. Genet..

[22] Sijtsma A., Rienks J., van der Harst P., Navis G., Rosmalen J.G.M., Dotinga A. (2021). Cohort Profile Update: Lifelines, a three-generation cohort study and biobank. Int. J. Epidemiol..

[23] Costa P.T., McCrae R.R. (1992). Revised NEO Personality Inventory (NEO-PI-R) and NEO Five-Factor Inventory (NEO-FFI).

[24] McCarthy S., Das S., Kretzschmar W., Delaneau O., Wood A.R., Teumer A., Kang H.M., Fuchsberger C., Danecek P., Sharp K. (2016). A reference panel of 64,976 haplotypes for genotype imputation. Nat. Genet..

[25] Neustaeter A., Nolte I., Snieder H., Jansonius N.M. (2021). Genetic pre-screening for glaucoma in population-based epidemiology: Protocol for a double-blind prospective screening study within Lifelines (EyeLife). BMC Ophthalmol..

[26] Auton A., Brooks L.D., Durbin R.M., Garrison E.P., Kang H.M., Korbel J.O., Marchini J.L., McCarthy S., McVean G.A., Abecasis G.R. (2015). A global reference for human genetic variation. Nature.

[27] Coombes B.J., Ploner A., Bergen S.E., Biernacka J.M. (2020). A principal component approach to improve association testing with polygenic risk scores. Genet. Epidemiol..

[28] Vinke P.C., Corpeleijn E., Dekker L.H., Jacobs D.R., Navis G., Kromhout D. (2018). Development of the food-based Lifelines Diet Score (LLDS) and its application in 129,369 Lifelines participants. Eur. J. Clin. Nutr..

[29] RIVM (2011). NEVO-Tabel. Dutch Food Composition Table—Version 3.

[30] Schofield W. (1985). Predicting basal metabolic rate, new standards and review of previous work. Hum. Nutr. Clin. Nutr..

[31] Kromhout D., Spaaij C.J.K., De Goede J., Weggemans R.M. (2016). The 2015 Dutch food-based dietary guidelines. Eur. J. Clin. Nutr..

[32] Amine E.K., Baba N.H., Belhadj M., Deurenberg-Yap M., Djazayery A., Forrestre T., Galuska D.A., Herman S., James W.P.T., M’Buyamba Kabangu J.R. (2003). Diet, Nutrition and the Prevention of Chronic Diseases. Am. J. Clin. Nutr..

[33] Wendel-Vos G.C.W., Schuit A.J., Saris W.H.M., Kromhout D. (2003). Reproducibility and relative validity of the short questionnaire to assess health-enhancing physical activity. J. Clin. Epidemiol..

[34] Byambasukh O., Snieder H., Corpeleijn E. (2020). Relation Between Leisure Time, Commuting, and Occupational Physical Activity With Blood Pressure in 125 402 Adults: The Lifelines Cohort. J. Am. Heart Assoc..

[35] Boulos M.I., Jairam T., Kendzerska T., Im J., Mekhael A., Murray B.J. (2019). Normal polysomnography parameters in healthy adults: A systematic review and meta-analysis. Lancet Respir. Med..

[36] Schweren L.J.S., Haavik J., Li L., Skretting-Solberg B., Larsson H., Hartman C.A. (2021). Drinking habits and executive functioning: A propensity score-weighted analysis of 78,832 adults. medRxiv.

[37] Akimova E.T., Breen R., Brazel D.M., Mills M.C. (2021). Gene-environment dependencies lead to collider bias in models with polygenic scores. Sci. Rep..

[38] Jaffee S.R., Price T.S. (2007). Gene–environment correlations: A review of the evidence and implications for prevention of mental illness. Mol. Psychiatry.

[39] Hunjan A.K., Hübel C., Lin Y., Eley T.C., Breen G. (2021). Association between polygenic propensity for psychiatric disorders and nutrient intake. Commun. Biol..

[40] Schweren L.J., Larsson H., Vinke P.C., Li L., Kvalvik L.G., Arias-Vasquez A., Haavik J., Hartman C.A. (2021). Diet quality, stress and common mental health problems: A cohort study of 121,008 adults. Clin. Nutr..

[41] Equivalence Factors for Disposable Household Income. https://longreads.cbs.nl/welvaartinnederland-2019/bijlagen/.

[42] Ganzeboom H.B.G. A New International Socio-Economic Index (ISEI) of Occupational Status for the International Standard Classification of Occupation 2008 (ISCO 08). Constructed with Data from the ISSP 2002–2007. Proceedings of the Paper presented at Annual Conference of International Social.

[43] van Buuren S., Groothuis-Oudshoorn K. (2011). Mice: Multivariate imputation by chained equations in R. J. Stat. Softw..

[44] Turley P., Walters R.K., Maghzian O., Okbay A., Lee J.J., Fontana M.A., Nguyen-Viet T.A., Wedow R., Zacher M., Furlotte N.A. (2018). Multi-trait analysis of genome-wide association summary statistics using MTAG. Nat. Genet..

[45] Grotzinger A.D., Rhemtulla M., de Vlaming R., Ritchie S.J., Mallard T.T., Hill W.D., Ip H.F., Marioni R.E., McIntosh A.M., Deary I.J. (2019). Genomic structural equation modelling provides insights into the multivariate genetic architecture of complex traits. Nat. Hum. Behav..

[46] Kale D., Stautz K., Cooper A. (2018). Impulsivity related personality traits and cigarette smoking in adults: A meta-analysis using the UPPS-P model of impulsivity and reward sensitivity. Drug Alcohol Depend..

[47] Stautz K., Cooper A. (2013). Impulsivity-related personality traits and adolescent alcohol use: A meta-analytic review. Clin. Psychol. Rev..

[48] Gillett G., Watson G., Saunders K.E., McGowan N.M. (2021). Sleep and circadian rhythm actigraphy measures, mood instability and impulsivity: A systematic review. J. Psychiatr. Res..

[49] Selinus E.N., Durbeej N., Zhan Y., Lichtenstein P., Lundström S., Ekblom M. (2021). Inattention and hyperactivity symptoms in childhood predict physical activity in adolescence. BMC Psychiatry.

[50] Smith K.E., Lavender J.M., Leventhal A.M., Mason T.B. (2021). Facets of Impulsivity in Relation to Diet Quality and Physical Activity in Adolescence. Int. J. Environ. Res. Public Health.

[51] Monroe S.M., Simons A.D. (1991). Diathesis-stress theories in the context of life stress research: Implications for the depressive disorders. Psychol. Bull..

[52] Brouwer-Brolsma E.M., Perenboom C., Sluik D., van de Wiel A., Geelen A., Feskens E.J., de Vries J.H. (2022). Development and external validation of the “Flower-FFQ”: A FFQ designed for the Lifelines Cohort Study. Public Health Nutr..

[53] Siebelink E., Geelen A., De Vries J.H.M. (2011). Self-reported energy intake by FFQ compared with actual energy intake to maintain body weight in 516 adults. Br. J. Nutr..

[54] Müller M., Kersten S. (2003). Nutrigenomics: Goals and strategies. Nat. Rev. Genet..

[55] Mondal S., Panda D. (2021). Nutrigenomics: An Interface of Gene-Diet-Disease Interaction. Mineral Deficiencies - Electrolyte Disturbances, Genes, Diet and Disease Interface.

[56] Bartke T., Schneider R. (2020). You are what you eat—How nutrition and metabolism shape the genome through epigenetics. Mol. Metab..

[57] Vucetic Z., Carlin J.L., Totoki K., Reyes T.M. (2012). Epigenetic dysregulation of the dopamine system in diet-induced obesity. J. Neurochem..

[58] Alsiö J., Olszewski P., Norbäck A., Gunnarsson Z., Levine A., Pickering C., Schiöth H. (2010). Dopamine D1 receptor gene expression decreases in the nucleus accumbens upon long-term exposure to palatable food and differs depending on diet-induced obesity phenotype in rats. Neuroscience.

