# Supplementary material for: Do Poor Diet and Lifestyle Behaviors Modify the Genetic Susceptibility to Impulsivity in the General Population?
Source: Nutrients. 2023 Mar 27;15(7):1625. doi: 10.3390/nu15071625 (PMC10096670; doi:10.3390/nu15071625)
Supplement: Supplementary file 1 [file nutrients-15-01625-s001.zip › nutrients-2274179 - supplementary.pdf]

Table S1. Pearson's correlations between the four indicators of dietary habits

	LLDS-I	KCAL	SUGAR	FAT
LLDS-I	1			
KCAL	0.185	1		
SUGAR	0.409	0.135	1	
FAT	0.253	0.208	-0.254*	1

LLDS-I represents the inverted Lifelines Diet Score, higher LLDS-I means poor overall diet quality; KCAL, Energy intake ratio; FAT, Fat intake ratio; SUGAR, Free sugar intake ratio.
* a negative correlation arises as both percentages are relative to total energy intake, i.e. they add up (together with percentage carbohydrates and percentage alcohol) to 100%.

Table S2. Gene-environment correlations between the PRS and diet/other lifestyles.

	Correlation r	P value
**Diet**		
Overall diet quality	0.015	0.006
Intake of energy	-0.031	1.14×10^{-8}
Intake of sugar	0.022	7.65×10^{-5}
Intake of fat	0.018	0.001
**Other lifestyle**		
MVPA	-0.002	0.740
Sleep hours	-0.014	0.012
Alcohol intake	0.007	0.189
Smoking	Beta	
Past	0.109	$<2\times10^{-16}$
Current	0.202	$<2\times10^{-16}$

MVPA indicates minutes per week spent in moderate-to-vigorous physical activity.

Table S3. Sensitivity analyses results of energy intake and its interaction with the PRS after including the interactions between energy intake and all four SES indices.

	B (95% CI)	*P* Value	Beta
**Intake of energy (KCAL) (ref=Q1)**			
KCAL_Q2	0.075 (-0.038, 0.189)	0.194	0.036
KCAL_Q3	0.148 (0.034, 0.263)	0.011	0.071
ADHD PRS	0.009 (-0.009, 0.026)	0.331	0.009
ADHD PRS×KCAL_Q2	0.032 (0.008, 0.056)	0.010	0.018
ADHD PRS×KCAL_Q3	0.036 (0.012, 0.061)	0.003	0.021

Q1indicates tertile 1; Q2, tertile 2; Q3, tertile 3. ADHD PRS×variable represents the interaction term between ADHD PRS and the variable.
